# The role of laboratory medicine specialists in the COVID-19 pandemic

**DOI:** 10.1515/almed-2020-0037

**Published:** 2020-05-18

**Authors:** Antonio Buño Soto

**Affiliations:** Department of Laboratory Medicine, Hospital Universitario La Paz, Paseo de la Castellana 261, Madrid, 28046, Spain

**Keywords:** coronavirus, COVID-19, laboratory medicine

The World Health Organization recently recognized the outbreak of the novel coronavirus SARS2-CoV as a pandemic, which has forced governments to issue a health alert about a disease that constitutes an international public health emergency. The disease caused by the coronavirus was first reported in Wuhan, Hubei, China by the end of December 2019, expanded rapidly throughout the country and spread to other Asian countries. At present, Europe and the United States have become the epicenter of the outbreak, with cases having been confirmed in almost all countries of the world [1].

Since the turn of the century, the world has witnessed several viral outbreaks that represented a global health threat. The first outbreak of pneumonia caused by a novel SARS coronavirus was declared in 2002. Some years later, in 2012, another new coronavirus caused an outbreak of severe respiratory infection in the Middle East (MERS Coronavirus). In 2014, the world faced the largest Ebola outbreak ever declared, which started in West Africa. These outbreaks led policymakers establish protocols for the management of patients with life-threatening infectious diseases that have turned to be extremely useful for the ongoing COVID-19 outbreak.

The rapid spread of this pandemic has changed our lives overnight in a way that was unimaginable some weeks ago, and it is challenging health systems worldwide. In these turbulent times, we have the obligation to do our best to care for the population in general and patients in particular.

As laboratory medicine specialists, we are aware of the challenges that our hospitals and clinics are facing these days. Since the beginning of the outbreak, laboratories have been crucial to the adequate organization of care. In this complex setting, the clinical laboratory is playing a pivotal role ranging from diagnosis of infection by the identification of the virus in airway specimens until the provision of evidence for adequate follow-up, prognosis, and decision making. In such a challenging context, health professionals are caring for their patients with absolute dedication, although some irrational behaviors have been observed as a result of a lack of understanding.

The COVID-19 epidemic has presented novel, numerous, and tough challenges to healthcare workers in a short period of time:

– A paradoxical situation has arisen where routine practice has almost stopped suddenly while the demand generated by COVID-19 has increased rapidly, up to the point that some hospitals provide care almost exclusively to coronavirus patients. This change of scenario has forced us to readjust healthcare pathways, protocols and staffs, considering that numerous healthcare professionals are becoming infected, which results in a shrinking of the workforce.
– The epidemic has forced us to reorganize our work and retask our laboratories in order to provide support to other units and departments. With great struggle and in record time, we have taken charge of the planning of the clinical laboratories of the field hospitals set up to support hospitals [2].
– Safety measures in clinical laboratories have been reinforced and lab staff had to learn how to use individual protection equipment. In addition, lab staff has been flooded with a plethora of protocols, recommendations and operating procedures that, far from being of help, have contributed to generate confusion and chaos among the staff in many cases.
– We have made a huge effort to learn about this new nosologic entity in a very short time. In some weeks, a myriad of publications have appeared in the scientific literature on the novel virus (about 1,500 as of March 28th, 2020 in Pubmed). This forced us to identify the most relevant ones and perform a review of publications to acquire knowledge of the disease, identify the most relevant evidence, and understand the need to quantify d-dimer, interleukin-6, ferritin, cardiac markers and perform yes/no infection tests in coronavirus patients. The evidence published also helps us understand the usefulness of working with diagnostic profiles and performing follow-up in the ICUs [3]. All this, on the awareness that our colleagues expect some help from us to mitigate uncertainty. Some of them have stopped providing their expertise as specialists to care for patients with the humility and savoir-faire needed to face this emergency situation.

The COVID-19 outbreak requires that leadership is exercised with determination at all levels, from health authorities to clinical laboratories. In a fast evolving environment, rapid – but not hurried – decisions have to be made. It is of paramount importance that decisions in the daily rush are made with the temperance required to be able to revise and reorganize units and laboratories and adapt them to changing needs and demands. Thoughtless action without reflection of its usefulness is not of help. Keeping a level head in this situation is crucial.

Our predecessors did not tell us how to face such a situation and we did not learn how to manage a pandemic at university. It is time that all health workers join forces to overcome this crisis. In this unprecedented situation, laboratory professionals will certainly get the most out of their savoir-faire and will have the ability to learn from their mistakes. This way, we will ensure that we are adequately prepared for the next pandemic.
